# Spontaneous spinal subarachnoid hemorrhage after severe coughing: a case report

**DOI:** 10.1186/1752-1947-7-274

**Published:** 2013-12-23

**Authors:** Yutaka Oji, Kazuyuki Noda, Joji Tokugawa, Kazuo Yamashiro, Nobutaka Hattori, Yasuyuki Okuma

**Affiliations:** 1 Department of Neurology, Juntendo University Shizuoka Hospital, 1129 Nagaoka, Izunokuni, Shizuoka 410-2295, Japan; 2 Department of Neurology, Juntendo University School of Medicine, 2-1-1 Hongo, Bunkyo, Tokyo 113-8421, Japan; 3 Department of Neurosurgery, Juntendo University Shizuoka Hospital, 1129 Nagaoka, Izunokuni, Shizuoka 410-2295, Japan

**Keywords:** Back pain, Headache, Spinal cord, Spinal subarachnoid hemorrhage, Spontaneous

## Abstract

**Introduction:**

Spinal subarachnoid hemorrhage has many causes including trauma, vascular malformations, aneurysms, spinal cord tumors, coagulation abnormalities, use of anticoagulants, systemic lupus erythematosus, or Behçet’s disease. We report on a rare case of a spontaneous spinal subarachnoid hemorrhage after severe coughing of unknown origin. To the best of our knowledge, this is the first report of spontaneous spinal subarachnoid hemorrhage after severe coughing.

**Case presentation:**

A 66-year-old Japanese woman initially complained of headache with severe back pain after severe coughing. She was referred to our neurology department 6 days after her first visit to our hospital. No neurological deficits were revealed except for meningism. Computed tomography of her head revealed no abnormality. A lumbar puncture showed bloody cerebrospinal fluid with xanthochromia. Cerebral angiography revealed no abnormality. Magnetic resonance imaging of her lumbar spine revealed subarachnoid hemorrhage. Spinal angiography revealed no abnormality. The diagnosis of spontaneous spinal subarachnoid hemorrhage was made. She recovered with conservative treatment and her neurological status was normal 2 years after the onset.

**Conclusions:**

Spontaneous spinal subarachnoid hemorrhage could be caused by rapid changes in intrathoracic and intra-abdominal pressure. Spontaneous subarachnoid hemorrhage should be considered when sudden back pain associated with severe headache develops. Even though emergent surgical decompression is necessary when the neurological state progressively deteriorates, conservative treatment with close monitoring of the symptoms can be recommended for patients with a stable neurological status.

## Introduction

Spinal subarachnoid hemorrhage and/or hematoma (SSH) is rare and represents less than 1% of all subarachnoid hemorrhage cases [1]. SSH is usually caused by several well-known predisposing factors, including trauma (often caused by lumbar puncture), coagulopathy, arteriovenous malformation, aneurysm, neoplastic lesions, systemic lupus erythematosus, and Behçet’s disease (BD) [2]. SSH may also occur spontaneously, which is extremely rare.

## Case presentation

**Figure 1:**
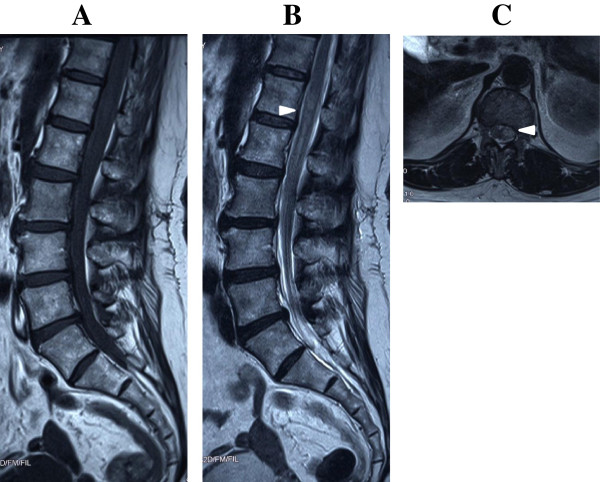
**Initial magnetic resonance imaging.** A sagittal T1-weighted image shows diffuse isosignal intensity in the subarachnoid space. No normal-appearing thecal sac or nerve roots are observed **(A)**. Sagittal T2-weighted **(B)** and axial T2-weighted **(C)** images show a high signal intensity from L1 to L2 (arrowheads).

**Figure 2:**
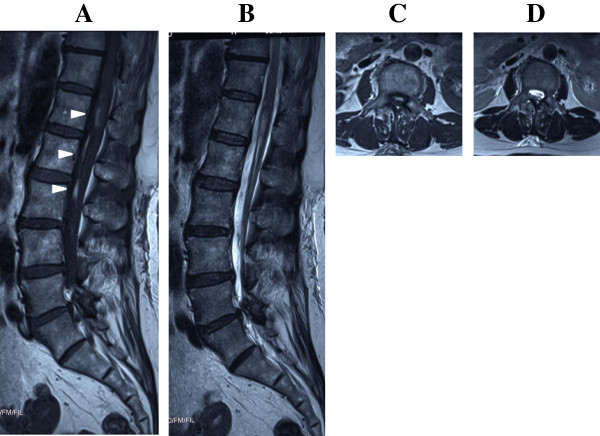
**Magnetic resonance imaging 8 days after onset.** Sagittal T1-weighted **(A)** and sagittal T2-weighted **(B)** images show subarachnoid hemorrhage extending from L1 to L3 ventrally to the spinal cord (arrowheads). There is no severe compression of the cord. The axial T1-weighted **(C)** and axial T2-weighted **(D)** images are at L1.

We here describe the case of a 66-year-old Japanese woman with a history of hypertension who presented with SSH after severe coughing of unusual spontaneous origin. She suddenly developed a severe headache accompanied by vomiting after severe coughing. Her headache was alleviated within approximately minutes, but severe back pain suddenly developed. She visited the orthopedic department of our hospital, and analgesic drugs were prescribed. Improvement of her back pain with analgesic drugs was temporary, and her headache with nausea exacerbated and became progressively worse. She visited the neurology department 6 days after the onset. Computed tomography of her brain showed no abnormal findings, but a lumbar puncture revealed bloody cerebrospinal fluid (CSF). She was immediately admitted to the neurology department of our hospital with the diagnosis of subarachnoid hemorrhage. She had no history of trauma and had not been prescribed anticoagulation agents. Her blood pressure on admission was 149/67mmHg. No neurological deficits were found except for meningism. There were no clinical features suggesting BD, including a positive pathergy test result or the presence of typical genital or ocular lesions. Hematological and coagulation function test results were normal. The test results of antinuclear antibodies and anti-double-stranded deoxyribonucleic acid were negative. Magnetic resonance (MR) images of her lumbar spine taken on day 1 were reviewed retrospectively. A sagittal T1-weighted MR image showed a diffuse isosignal intensity in the subarachnoid space; therefore, no normal-appearing thecal sac or nerve roots were observed (Figure 1A). Sagittal and axial T2-weighted MR images showed a high signal intensity, which was slightly lower than the signal intensity of CSF *per se*, from L1 to L2, in the ventrolateral subarachnoid space (Figure 1B and 1C). These findings were indicative of acute hemorrhage. Digital subtraction angiography on day 7 disclosed no abnormal findings. MR images of the lumbar spine on day 8 showed high signal intensity on T1-weighted images, and a low signal intensity on T2-weighted images from L1 to L3, which was indicative of early subacute hemorrhage (Figure 2). No MR imaging evidence of vascular abnormalities was detected in her entire spine. MR images of her entire spine on day 8 showed no tumor-like staining by gadolinium on T1-weighted images. Although the patient was advised to undergo spinal angiography, she did not consent to it. The patient was discharged 25 days after admission without any neurological deficits. Spinal angiography was performed with her consent 2 months after the onset, which disclosed no abnormal findings. The diagnosis of spontaneous SSH was confirmed. Repeated MR imaging showed no recurrence, and no signal change within the spinal cord. She was healthy at the 2-year follow-up examination.

## Discussion

The symptoms of this patient were sudden-onset severe headache and back pain. Although the sudden onset of severe back pain is a characteristic symptom of SSH, it is difficult to distinguish between epidural, subdural, and subarachnoid spinal hemorrhage [3-6]. It has been estimated that 80% of patients with SSH have concomitant intracranial symptoms such as headache (70%) and mental changes (22%) [7]. The headache of this patient was associated with meningism, and spinal tapping revealed bloody CSF. These findings strongly suggested that the origin of her hemorrhage was in the subarachnoid space.

Spontaneous SSH is rare. To the best of our knowledge, only 20 individual cases of spontaneous SSH, including our patient without any apparent source of bleeding, have been reported [1-6,8-15]. The paroxysmal onset of severe back pain and headache is observed in 11 of 20 spontaneous SSH cases, including our patient [2-4,6,8-10,12,14,15]. Consciousness disturbance was observed in three of the 20 cases [3,10]. The circumstances at the time of onset of symptoms include eating, bending the head, scuba diving, having sexual intercourse, defecating, receiving the recoil of a shotgun, and jumping into the sea [1-3,5,10,12,13,15]. To the best of our knowledge, there have been no reported cases of SSH that developed after severe coughing. With regard to the pathogenesis of the condition, it is considered that a forgotten effort or minor trauma increases intrathoracic and intra-abdominal pressure, and the intraluminal pressure of spinal vessels, particularly the valveless radiculomedullary veins crossing the subarachnoid space, which results in subsequent tearing of vessels within the subarachnoid space [2]. On the basis of this mechanism, we considered that the vessels within the subarachnoid space of the lumbar spine ruptured, which resulted in SSH because of a sudden increase in intra-abdominal pressure after the severe coughing in our patient.

Acute spontaneous SSH is a potentially dangerous condition and may have disastrous consequences; thus, urgent decompressive surgery should be performed when the neurological state progressively deteriorates, unlike in our patient [3,4,10-13]. When bleeding occurs in the subarachnoid space, the CSF may dilute the subarachnoid hemorrhage, and defibrination by pulsation of the spinal cord reduces the likelihood of subarachnoid hematoma formation [1,2]. In this patient, it is considered that these mechanisms effectively worked owing to the absence of mechanical obstacles within the spinal column such as spondylosis, disk herniation, and thickening of the yellow ligament. Komiyama *et al.* postulated that the ventral-type SSH causes acute back pain and minimal neurological deficits, and could be treated conservatively. Conversely, the dorsal type may require surgical intervention [2]. According to this theory, this patient had ventral SSH without neurological deficits, and could be managed conservatively. However, Ruelle *et al.* reported that in their patients the hemorrhage was located dorsally and ventrally, but they recovered without surgical decompression [9]. Therefore, the location of the hemorrhage does not seem to be the only factor deciding the treatment. It is considered that the decision to perform surgical decompression in SSH does not necessarily depend on the hemorrhage location, but on the neurological status of the patient [1].

## Conclusions

We suggest that spontaneous SSH should be considered when sudden back pain associated with severe headache develops. Spontaneous SSH may resolve with conservative treatment with close patient monitoring, when the patient has no neurological deficits. However, we should pay close attention to the progression of the neurological symptoms, and appropriate patient triage and timely neurosurgical intervention should be considered.

## Consent

Written informed consent was obtained from the patient for the publication of this case report and its accompanying images. A copy of the written consent is available for review by the Editor-in-Chief of this journal.

## Competing interests

The authors declare that they have no competing interests.

## Authors’ contributions

YO, KN, JT, KY, and YO cared for the patient in both the in-patient and out-patient settings. NH reviewed the manuscript and provided suggestions. All the authors contributed to the writing of the manuscript and read and approved the final version of the manuscript.
